# Psyllids, It’s What’s on the Inside That Counts: Community Cross Talk Facilitates Prophage Interactions

**DOI:** 10.1128/mSphere.00227-17

**Published:** 2017-06-07

**Authors:** Allison K. Hansen, Isabel H. Skidmore

**Affiliations:** Department of Entomology, University of Illinois, Urbana-Champaign, Urbana, Illinois, USA

**Keywords:** Liberibacter, SC-1, *Wolbachia*, community cross talk, endosymbiont, prophage, psyllid, symbiosis

## Abstract

Despite the availability of massive microbial community data sets (e.g., metagenomes), there is still a lack of knowledge on what molecular mechanisms facilitate cross talk between microbes and prophage within a community context. A study published in *mSphere* by Jain and colleagues (M. Jain, L. A. Fleites, and D. W. Gabriel, mSphere 2:e00171-17, 2017, https://doi.org/10.1128/mSphereDirect.00171-17) reports on an intriguing new twist of how a prophage of the bacterium “*Candidatus* Liberibacter asiaticus” may have its lytic cycle suppressed partly because of a protein that is expressed by a cooccurring bacterium, *Wolbachia*. Both of these microbes coexist along with other microbial tenants inside their sap-feeding insect host, a psyllid. Although these results are still preliminary and alternative hypotheses need to be tested, these results suggest an interesting new dimension on how regulation of microbial genomes occurs in a community context.

## Abstract (precis)

Despite the availability of massive microbial community data sets (e.g., metagenomes), there is still a lack of knowledge on what molecular mechanisms facilitate cross talk between microbes and prophage within a community context.

## COMMENTARY

The effect of microbes on animals and plants is a field of study reaching its prime. Yet, our limited awareness of the diversity of mechanisms that facilitate direct and indirect cross talk among community members is evident. There is a wealth of knowledge on microbe-eukaryote interactions, especially from a pathogenic angle; however, our current framework for understanding multidomain phage interactions is largely descriptive at this time. In their article, Jain et al. (1) go beyond description, reporting on the molecular mechanisms of a new type of cross talk involving a community of bacteria and a phage within an insect host.

Microbial communities associated with animals can be highly diverse and dynamic, especially in vertebrate guts. In contrast, these microbial communities are less diverse and more stable inside arthropod bodies, a fact exemplified by sap-feeding insects. For example, sap-feeding insects are known to harbor endosymbiotic bacteria that are vertically transmitted and that are either obligate or facultative for insect survival (2). These insects can also harbor bacteria that circulate throughout the insect’s body, which do not cause morbidity to the insect host but instead are vectored to their food plants as plant pathogens (3). In their article, Jain et al. (1) examine one of these simple microbial communities residing inside the sap-feeding insect *Diaphorina citri*, a global psyllid pest of citrus. This psyllid’s status as a pest results from its ability to vector the deadly citrus pathogen, “*Candidatus* Liberibacter asiaticus” (4). This insect also harbors two obligate nutritional endosymbionts (5, 6) and a facultative endosymbiont, *Wolbachia*. Thus, after feeding on an infected plant, “*Ca.* Liberibacter asiaticus” establishes a circulative infection in psyllids, resulting in four distinct microbes colonizing the insect. The significance of Jain et al.’s study (1) lies in their suggestion that *Wolbachia* expresses a protein potentially able to enter through the cell membrane of “*Ca.* Liberibacter asiaticus” during coinfection. Interestingly, this *Wolbachia* gene-encoded protein may play a major role in repressing the promoter region of a lytic prophage gene, holin, that is encoded in the genome of “*Ca.* Liberibacter asiaticus.” The holin promoter is at the start of an operon for several lytic genes (7), and therefore, the promoter region may be a major switch that represses the lytic stage of prophage SC-1. This is particularly interesting from a community perspective: lytic genes associated with this prophage are highly expressed when “*Ca.* Liberibacter asiaticus” infects citrus, whereas these genes are repressed in the psyllid’s body. These data suggest that the lytic life cycle, while induced in the stressful host plant environment, is repressed in the insect vector in part by microbial community members (1). This is also interesting from a pest control perspective as the psyllid’s vector competence for “*Ca.* Liberibacter asiaticus” may be reduced if the *Wolbachia* repressor protein can be silenced or edited.

Jain et al. (1) initially tease apart the molecular mechanisms of this community cross talk by first isolating and identifying the putative repressor protein from an extract of the insect’s cell contents. This extract was previously revealed to repress promoter activity of the lytic prophage holin gene in a dose-dependent manner (8) and contains a community of cells from not only the psyllid itself but also from the obligate and facultative endosymbionts of this psyllid in addition to “*Ca.* Liberibacter asiaticus.” Using a pulldown assay followed by a mass spectrometry approach, Jain et al. (1) revealed that the best candidate for this repressor protein was a small 56-amino-acid hypothetical *Wolbachia* protein that does not have any significant homology to other known proteins. Because “*Ca.* Liberibacter asiaticus” is not yet culturable, the relative *Liberibacter crescens* was used as a genetic model to further demonstrate that the putative *Wolbachia* repressor protein can enter into bacterial cells similar to “*Ca.* Liberibacter asiaticus.” The *Wolbachia* protein was indeed found to repress the promoter of the holin gene, albeit not in a dose-dependent manner (1). The authors note that these results, in conjunction with the fact that insect extract preparations that were heated and treated with proteinase K displayed reduced repressor activity, suggest that other unknown proteins and/or protein modifications may be necessary for the complete suppression of the holin promoter.

While these findings hold promise, an important alternative mechanism not addressed by Jain et al. (1) is the importance of the prophage’s own repressor protein in maintaining the lysogenic cycle inside the insect host. As a dose-dependent response was achieved with the psyllid extract but not with the *Wolbachia* protein alone, the prophage SC-1 gene-encoded repressor may be the missing puzzle piece to understanding prophage regulation. Prophage SC-1 encodes a P22 C2-like helix-turn-helix prophage repressor gene along with a putative Bro-N family antirepressor gene (7). Thus, SC-1 appears to encode regulatory circuitry analogous to the repressor gene *c*I and the antirepressor gene *cro* of phage lambda, the classic genetic model for temperate prophages. During lysis, phage expresses a repressor gene that enables transcriptional repression of the antirepressor gene along with other genes involved in the lytic cycle. However, when the bacterial host is subjected to stress (e.g., “*Ca.* Liberibacter asiaticus” infecting citrus), the repressor protein is outcompeted by the antirepressor, thereby silencing expression of the repressor protein and turning on the lytic cycle (9). While Jain et al.’s data (1) indicate that the *Wolbachia* protein may play a role in reinforcing the lysogenic cycle, innate regulation by prophage SC-1 should be investigated. Additionally, expression of this *Wolbachia* protein does not appear to be specific to the presence of “*Ca.* Liberibacter asiaticus,” as it is constitutively expressed whether the psyllid is infected with “*Ca.* Liberibacter asiaticus” or not (1). This weakens Jain et al.’s argument (1) that this interaction is an adaptive response of *Wolbachia* to avoid the psyllid’s immune response to the lytic cycle of “*Ca.* Liberibacter asiaticus.” Nevertheless, the possibility that this *Wolbachia* protein can translocate across the cell membrane of “*Ca.* Liberibacter asiaticus” and control the expression of prophage genes is intriguing and should be investigated further with microscopic localization approaches *in vivo*.

A previous study by Nakabachi and colleagues (10) demonstrated that a protein of bacterial origin can translocate across an endosymbiont’s cell membrane within the pea aphid. This study contrasts with Jain et al.’s study (1), in that the bacterial protein was horizontally transferred into the aphid’s genome and expressed from the insect’s cell, rather than from another bacterial cell. Cross talk among phages, bacteria, and insects has also been characterized previously in *Wolbachia* phage WO (11) and “*Candidatus* Hamiltonella defensa” phage APSE (12); however, the exact molecular mechanisms facilitating cross talk in these systems, especially with insects, remain enigmatic. Mixed-domain cross talk via RNAs has also been described: *Wolbachia-*expressed small RNAs were found to impact insect gene expression (13). Genome-enabled research on nonculturable obligate symbiont systems further corroborates the potential for cooccurring endosymbionts and insect hosts to exchange proteins and/or intermediates to complement pathways for the biosynthesis of essential nutrients that are incomplete in the endosymbionts’ genomes (2, 14, 15). As biotechnology and computational approaches improve, our ability to discover more about the molecular mechanisms that govern these community interactions will increase, and the web of community cross talk will slowly be untangled.

## References

[1] Jain M, Fleites LA, Gabriel DW 2017 A small *Wolbachia* protein directly represses phage lytic cycle genes in “*Candidatus* Liberibacter asiaticus” within psyllids. mSphere 2:e00171-17. doi:10.1128/mSphere.00171-17. PMC5463029 28608866

[2] Hansen AK, Moran NA 2014 The impact of microbial symbionts on host plant utilization by herbivorous insects. Mol Ecol 23:1473–1496. doi:10.1111/mec.12421. 23952067

[3] Casteel CL, Hansen AK 2014 Evaluating insect-microbiomes at the plant-insect interface. J Chem Ecol 40:836–847. doi:10.1007/s10886-014-0475-4. 25052911

[4] Grafton-Cardwell EE, Stelinski LL, Stansly PA 2013 Biology and management of Asian citrus psyllid, vector of the huanglongbing pathogens. Annu Rev Entomol 58:413–432. doi:10.1146/annurev-ento-120811-153542. 23317046

[5] Nakabachi A, Ueoka R, Oshima K, Teta R, Mangoni A, Gurgui M, Oldham NJ, van Echten-Deckert G, Okamura K, Yamamoto K, Inoue H, Ohkuma M, Hongoh Y, Miyagishima SY, Hattori M, Piel J, Fukatsu T 2013 Defensive bacteriome symbiont with a drastically reduced genome. Curr Biol 23:1478–1484. doi:10.1016/j.cub.2013.06.027. 23850282

[6] Nakabachi A, Yamashita A, Toh H, Ishikawa H, Dunbar HE, Moran NA, Hattori M 2006 The 160-kilobase genome of the bacterial endosymbiont *Carsonella*. Science 314:267. doi:10.1126/science.1134196. 17038615

[7] Zhang S, Flores-Cruz Z, Zhou L, Kang BH, Fleites LA, Gooch MD, Wulff NA, Davis MJ, Duan YP, Gabriel DW 2011 ‘*Ca*. Liberibacter asiaticus’ carries an excision plasmid prophage and a chromosomally integrated prophage that becomes lytic in plant infections. Mol Plant Microbe Interact 24:458–468. doi:10.1094/MPMI-11-10-0256. 21190436

[8] Fleites LA, Jain M, Zhang S, Gabriel DW 2014 “*Candidatus* Liberibacter asiaticus” prophage late genes may limit host range and culturability. Appl Environ Microbiol 80:6023–6030. doi:10.1128/AEM.01958-14. 25063651 PMC4178692

[9] Court DL, Oppenheim AB, Adhya SL 2007 A new look at bacteriophage genetic networks. J Bacteriol 189:298–304. doi:10.1128/JB.01215-06. 17085553 PMC1797383

[10] Nakabachi A, Ishida K, Hongoh Y, Ohkuma M, Miyagishima SY 2014 Aphid gene of bacterial origin encodes a protein transported to an obligate endosymbiont. Curr Biol 24:R640–R641. doi:10.1016/j.cub.2014.06.038. 25050957

[11] Kent BN, Bordenstein SR 2010 Phage WO of *Wolbachia*: lambda of the endosymbiont world. Trends Microbiol 18:173–181. doi:10.1016/j.tim.2009.12.011. 20083406 PMC2862486

[12] Oliver KM, Degnan PH, Burke GR, Moran NA 2010 Facultative symbionts in aphids and the horizontal transfer of ecologically important traits. Annu Rev Entomol 55:247–266. doi:10.1146/annurev-ento-112408-085305. 19728837

[13] Mayoral JG, Hussain M, Joubert DA, Iturbe-Ormaetxe I, O’Neill SL, Asgari S 2014 *Wolbachia* small noncoding RNAs and their role in cross-kingdom communications. Proc Natl Acad Sci U S A 111:18721–18726. doi:10.1073/pnas.1420131112. 25512495 PMC4284532

[14] McCutcheon JP, Moran NA 2011 Extreme genome reduction in symbiotic bacteria. Nat Rev Microbiol 10:13–26. doi:10.1038/nrmicro2670. 22064560

[15] Skidmore IH, Hansen AK 29 3 2017 The evolutionary development of plant-feeding insects and their nutritional endosymbionts. Insect Sci https://doi.org/10.1111/1744-7917.12463. 10.1111/1744-7917.12463 28371395

